# AKI and Urticaria in a Kidney Transplant Patient Undergoing Therapeutic Apheresis

**DOI:** 10.34067/KID.0000000000000319

**Published:** 2024-03-26

**Authors:** Nikhil A. Reddy, Ashraf I. Reyad, Sridhar R. Allam

**Affiliations:** 1 North Texas Division, HCA Healthcare Research Institute, Fort Worth, Texas; 2 Transplant Surgery, Medical City Fort Worth Transplant Institute, Fort Worth, Texas; 3 Transplant Nephrology, PPG Health, Fort Worth, Texas

**Keywords:** acute rejection, kidney transplantation, renal ischemia

## Abstract

**Figure:**
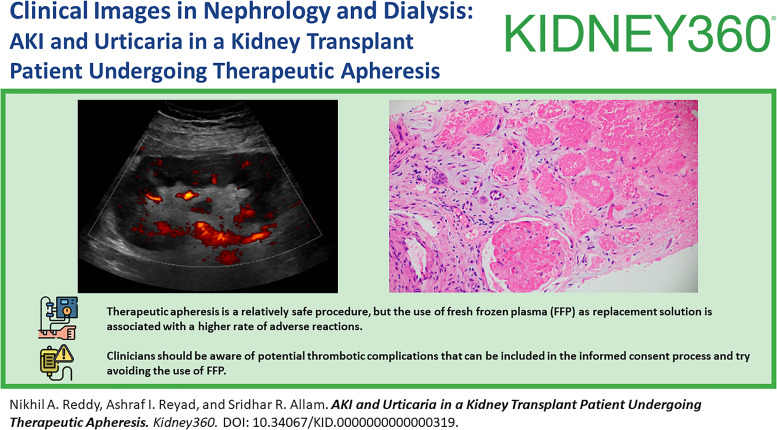

## Case Description

**Figure 1:**
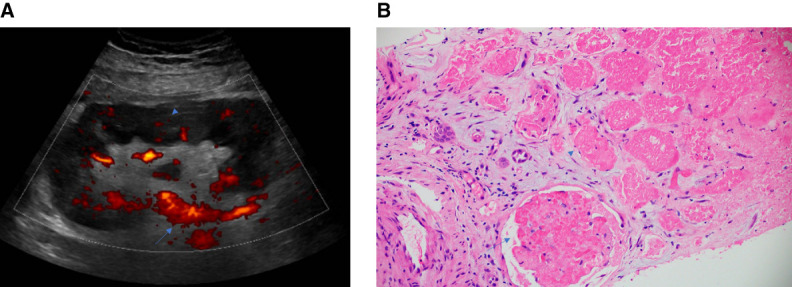
**Kidney transplant ultrasound and biopsy showing microvascular thrombosis.** (A) Kidney transplant ultrasound showing patent main artery (arrow) but poor intraparenchymal perfusion (arrowhead). (B) Kidney transplant biopsy hematoxylin and eosin stain showing infarction of about 50% of the cortex involving glomerulus (arrow) and tubulointerstitial area (arrowhead).

A 51-year-old man with ESKD due to polycystic kidney disease received a deceased donor kidney transplant. Seven months post-transplant, serum creatinine (sCr) increased to 1.8 mg/dl from a baseline of 1.3 with the development of donor-specific antibody against DQ7 at a mean fluorescence intensity of 15,506. Donor-derived cell-free DNA was noted to be 4.7%. Biopsy revealed acute antibody-mediated rejection (AMR). After three doses of methylprednisolone, two doses of bortezomib, and two sessions of therapeutic apheresis (TA), sCr improved to 1.3. For the third session of TA, fresh frozen plasma (FFP) was used as replacement solution because of the risk of bleeding at the recent kidney transplant biopsy site with a decrease of fibrinogen to 94 mg/dl after first two sessions of TA. During this session, the patient developed acute respiratory distress with urticaria and diaphoresis requiring termination of TA. Intravenous diphenhydramine and methylprednisolone were administered with gradual improvement in symptoms. X-ray of the chest revealed clear lung fields. Workup for acute hemolytic transfusion reaction was negative. The following morning, sCr doubled to 2.6 with leukocytosis (white blood cell count, 11.6 → 31.0), thrombocytopenia (platelet count, 155 → 103), transaminitis (aspartate aminotransferase, 15 → 214; alanine aminotransferase, 16 → 157), and a very high lactate dehydrogenase of 2235 units/L. Partial thromboplastin time was 32.9 seconds, and international normalized ratio was 1.4. Donor-specific antibody improved to a mean fluorescence intensity of 7204. Kidney transplant ultrasound showed patent main artery and vein but poor intraparenchymal perfusion (Figure 1A). No hypotension or other thromboembolic manifestations were noted during his course. He had a negative hypercoagulability panel at the time of transplant evaluation (done as part of our center protocol for all wait list patients). sCr further worsened to 4.5, 5.3, and 5.9, respectively, on the following consecutive days. He received two more sessions of TA using 5% albumin as replacement solution and a dose of rituximab after last TA along with increased maintenance immunosuppressive therapy for AMR. sCr eventually stabilized around 4.5 and donor-derived cell-free DNA improved to 0.09%. Follow-up kidney transplant biopsy about 4 weeks after the initial one revealed no active AMR but infarction involving about 50% of the cortex (Figure 1B).

## Discussion

TA is frequently used in the management of AMR after kidney transplant. TA can result in several adverse effects. Although native kidney thrombosis due to TA was reported previously in a patient with Guillain-Barre syndrome,^1^ kidney transplant infarction has not been previously reported. In a study of 20 kidney transplant recipients with AMR treated with plasmapheresis, two patients developed allograft infarction at diagnosis before treatment was initiated, and two more patients developed allograft infarction while on treatment.^2^ These were attributed to severe AMR, unlike in our patient who had mild AMR at diagnosis with improvement in sCr while on treatment but developed acute allograft dysfunction after the third session of TA. Serial TA is known to deplete several factors involved in the coagulation cascade. While bleeding complications from depletion of fibrinogen are feared more, depletion of antithrombin III can lead to thrombotic phenomena.^3^ Utilization of FFP as replacement solution for bleeding risk at the biopsy site likely had exacerbated the prothrombotic state in our patient. Another plausible mechanism for our patient's kidney allograft microvascular thrombosis could be related to anaphylactic reaction that he experienced during the third session of TA. Anaphylaxis was reported to be associated with the risk of thrombosis by activation of the contact and coagulation systems *via* factor XII and platelet-activating factor.^4,5^

## Teaching Points

- TA is a relatively safe procedure, but the use of FFP as replacement solution is associated with a higher rate of adverse reactions.
- Clinicians should be aware of potential thrombotic complications that can be included in the informed consent process and try avoiding the use of FFP.
